# Supplementary material for: SUV39 SET domains mediate crosstalk of heterochromatic histone marks
Source: eLife. 2021 Sep 15;10:e62682. doi: 10.7554/eLife.62682 (PMC8443253; doi:10.7554/eLife.62682)
Supplement: Supplementary file 1. — (A) Oligonucleotides used in qPCR experiments in Figure 4. (B) Peptide sequences used for synthesis of ubiquitinated H3 substrates. (C) sgRNA sequences used for CRISPR mutagenesis. [file elife-62682-supp1.docx]

## Supplementary file 1

### A) Oligonucleotides

| **Name** | **Construct** | **Sequence** | **Use** |
| --- | --- | --- | --- |
| O.1I0 | tlh1_R | CTCCTTGGAAGAATTGCAAGCCTC | qPCR Fig. 1F, 3D |
| O.1HZ | tlh1_F | ATGGTCGTCGCTTCAGAAATTGC | qPCR Fig. 1F, 3D |
| O.1HY | cen-dg_R | TGCTTCACGGTATTTTTTGAAATC | qPCR Fig. 1F, 3D |
| O.1HX | cen-dg_F | AAGGAATGTGCCTCGTCAAATT | qPCR Fig. 1F, 3D |
| O.1HW | cen-dh_F | GTATTTGGATTCCATCGGTACTATGG | qPCR Fig. 1F, 3D |
| O.1HV | cen-dh_R | ACTACATCGACACAGAAAAGAAAACAA | qPCR Fig. 1F, 3D |
| O.10Z | act1_R | TCTTTTCCATATCATCCCAGTTG | qPCR Fig. 1F, 3D |
| O.10Y | act1_F | CTCAAAGCAAGCGTGGTATTT | qPCR Fig. 1F, 3D |

### B) Peptides

Sequence of peptides synthesized by Fmoc-SPPS.

| **Name** | **Synthetic peptide sequence** |
| --- | --- |
| P1 | Fmoc-ARTKQTARKSTGGK(alloc)APR-NHNH2 |
| P2 | Fmoc-ARTKQTARKSTGGKAPRK(alloc)QLA-NH2 |

### C) CRISPR sgRNAs

Sequence of sgRNA used in this study.

| **Name** | **Target** | **Sequence** |
| --- | --- | --- |
| sgRNA_1 | Clr4 | GACGAGAAATGAAGTGAAGG |
| sgRNA_2 | Clr4 | GCGAATGTCGCCTAAACAAG |
| sgRNA_3 | Clr4 | AGCCAGTACCGCTTAACACA |
| sgRNA_4 | Clr4 | GATTGAAAATTTGGATCAGG |
